# Early stimulation for neuropsychomotor development in children with microcephaly: a systematic review

**DOI:** 10.1590/1984-0462/2024/42/2023063

**Published:** 2023-12-18

**Authors:** Gabrielle Mascarenhas Canto, Katia de Miranda Avena

**Affiliations:** a Faculdade Zarns, Curso de Medicina, Salvador, BA, Brazil.

**Keywords:** Microcephaly, Child, Growth and development, Early intervention, Microcefalia, Criança, Crescimento e desenvolvimento, Intervenção precoce

## Abstract

**Objective:**

To systematically review studies on the effects of early stimulation on the neuropsychomotor development of children with microcephaly.

**Data source:**

A systematic review was conducted in PubMed/MEDLINE, Virtual Health Library, and Cochrane Library databases. Studies that addressed the use of early stimulation in playful and interactive environments in children with microcephaly were included. There were no restrictions on the publication date or language of the studies. The outcomes assessed were muscle tone, social interaction, fine and gross motor skills, intelligence quotient, socioemotional and adaptive behavior of the child. The methodological quality and the scientific evidence level were assessed using the Risk of Bias in Non-randomized Studies of Interventions, the Revised Cochrane risk of bias tool for randomized trials and the Grading of Recommendations Assessment, Development and Evaluation.

**Data synthesis:**

264 articles were identified, but only 7 met the eligibility criteria. The included studies had a total population of 125 individuals, with sample sizes ranging from 1 to 71 participants.

**Conclusions:**

The studies showed low evidence of an effect of early intervention on the outcomes muscle tone, social interaction, fine and gross motor skills, intelligence quotient, and socioemotional and adaptive behavior in children with microcephaly. However, further randomized clinical trials are needed.

## RESUMO

**Objetivo:**

Revisar, de forma sistemática, estudos sobre a interferência da estimulação precoce no desenvolvimento neuropsicomotor de crianças com microcefalia.

**Fontes de dados:**

Trata-se de uma revisão sistemática realizada nas bases de dados *United States National Library of Medicine/ Medical Literature Analysis and Retrieval System Online* (PubMed/MEDLINE), Biblioteca Virtual em Saúde (BVS) e Cochrane Library. Foram incluídos estudos que abordassem o uso de estímulo precoce em ambientes lúdicos e interativos em crianças com microcefalia. Não foram impostas restrições quanto à data de publicação e ao idioma dos estudos. Como desfechos, avaliaram-se tônus muscular, interação social, habilidades motoras fina e grossa, quociente de inteligência, comportamento socioemocional e adaptativo da criança. A qualidade metodológica dos estudos e o nível de evidência científica foram avaliados pelo *Risk of bias in non-randomized studies of interventions, Revised Cochrane risk of bias tool for randomized trials* e *Grading of recommendations assessment, development and evaluation*.

**Síntese dos dados:**

Foram identificados 264 artigos, entretanto apenas sete atenderam aos critérios de elegibilidade. Os estudos incluídos totalizam uma população de 125 indivíduos, com amostras que variaram de um a 71 participantes.

**Conclusões:**

Os estudos demonstraram a existência de baixa evidência quanto à interferência da intervenção precoce nos desfechos tônus muscular, interação social, habilidades motoras finas e grossas, quociente de inteligência e comportamento socioemocional e adaptativo em crianças com microcefalia. No entanto, novos ensaios clínicos randomizados ainda são necessários.

## INTRODUCTION

Microcephaly is a congenital malformation in which the child’s brain does not develop properly, causing babies to be born with a smaller head circumference than expected for their age, gender, and race.^1^ This congenital malformation can be caused by chemical substances and biological agents (such as bacteria and viruses) as well as exposure to radiation.^2^

In 2016, the World Health Organization (WHO) declared a state of international emergency due to the increased incidence of microcephaly in endemic areas with Zika virus proliferation.^3,4^ The disease, whose main vector is the Aedes aegypti mosquito, has spread rapidly in the Northeast region of Brazil, mainly due to the high summer temperatures in the southern hemisphere.^3^ Later, studies confirmed this positive association between the microcephaly outbreak in the Northeast region of the country and maternal contamination by Zika virus.^2,3–6^

The consequences of Zika virus contamination during pregnancy are irreversible,^7^ therefore, the earlier pregnant women are infected, the higher is the chance of damage to the neuropsychomotor development of the baby.^8,9^ Nonetheless, it is important to emphasize that there is no specific treatment for microcephaly. What do exist are support actions that can help the development of babies and children. As each child develops different complications (respiratory, neurological, and motor), monitoring by different specialists may be necessary, depending on the functions compromised.^10^

In this context, considering that there is no specific treatment for this malformation, early rehabilitation has been established as a useful therapeutic strategy to assist in the neuropsychomotor development of children with microcephaly. Early stimulation plays a fundamental role in the neuropsychomotor development of children, promoting the acquisition of essential motor, cognitive, and socioemotional skills during the early years of life.^1,10^ However, there is a significant knowledge gap regarding its effectiveness in children with microcephaly.

Given the importance of this therapeutic strategy, this study systematically analyzed the influence of early stimulation in the neuropsychomotor development of children with microcephaly.

## METHOD

A systematic literature review was performed using the Preferred Reporting Items for Systematic Reviews and Meta-Analyses (PRISMA) methodology. The research protocol was registered with the International Prospective Register of Systematic Reviews (PROSPERO) under registration number CRD42020173572.

The research question that guided the search strategy was: “Does the application of early stimulation in playful and interactive environments in children with microcephaly improve neuropsychomotor development?”.

The survey of scientific articles was carried out in the following databases and portals: MEDLINE^®^ via PubMed^®^, Virtual Health Library (VHL) and Cochrane Library, using specific search strategies for each of these databases (Table 1).

**Table 1:** Search strategy used in the systematic review.

Databases	Search strategy
MEDLINE^^®^^ via PubMed^^®^^	((child) AND microcephaly) AND Early Intervention, Educational ((child) AND Microcephaly) AND growth & development AND Cognition
Virtual Health Library	(child OR children) AND (microcephaly) AND (rehabilitation) {[Child OR Children] AND Microcephaly AND Early Intervention, Educational}
Cochrane Library	Microcephaly AND Child

Based on health sciences descriptors (DECs and MeSH), the following keywords were identified: Microcephaly; Growth and Development; Early Intervention, Educational; Occupational Therapy; Rehabilitation. Initially, broader terms were intentionally used in order to identify a greater number of studies that addressed the topic of interest and to minimize the chance that an important article would be excluded from this review. In addition, the reference lists of the primary studies included in the search were reviewed to identify possible studies that met the inclusion criteria, but that for some reason were not identified by the defined search strategy.

Prospective and retrospective studies that addressed the use of early stimulation in playful and interactive environments in children with microcephaly were included. No restrictions were placed on the publication date or language of the studies. Books, book chapters, editorials, and other text formats that had not undergone a rigorous peer review process similar to that for scientific articles were excluded. In addition, duplicate texts found by indexing in more than one database were excluded.

Outcomes of interest were muscle tone, social interaction, fine and gross motor skills, intelligence quotient, social-emotional and adaptive behavior of the child with the use of early stimulation.

Literature data collection was conducted between August 2020 and January 2023, with the last search date being January 31^st^, 2023. Once the studies were identified, two independent reviewers (G.M.C and K.M.A) performed the analysis. This step consisted of reading and analyzing the titles, abstracts, and keywords of the identified studies. At this point, review studies and those that did not meet the purpose of this study were excluded. After initial screening, the next step was to read the studies in their entirety. Finally, the studies were organized into tables to present their main information and to facilitate a descriptive and critical analysis of the results obtained by the authors.

The Risk of Bias in Non-randomized Studies of Interventions (ROBINS-I)^11^ was used to assess the risk of bias in the selected observational studies. The tool assesses seven domains of bias (confounding, selection, classification of interventions, departures from proposed interventions, information, outcome measurement, and selective reporting of results) and classifies them as low, moderate, high, or critical risk. The overall risk of the studies was then classified as low (low risk in all areas), moderate (moderate risk in at least one area) or severe (severe risk in at least one area). The assessment was performed by two independent reviewers (G.M.C and K.M.A). There were no cases of disagreement that required a third assessor. Interrater agreement was not measured in this study. Risk of bias VISualization^®^ (Robvis) was used to graphically display the risk of bias of the studies.

For experimental trials, the risk of bias was assessed using the Revised Cochrane risk of bias tool for randomized trials (RoB 2).^12^ The tool assesses five domains related to the risk of bias of the included clinical trials (randomization, departures from the intended interventions, loss of outcome data, outcome measurement, and outcome reporting). After analyzing the domains, the studies were classified as “high risk”, “some concern” or “low risk”. Again, the assessment was performed by two independent reviewers (G.M.C and K.M.A), with no disagreement between them. Interrater agreement was not measured.

The Grading of Recommendations Assessment, Development and Evaluation (GRADE)^13^ system was used to classify the quality of scientific evidence for the outcomes analyzed. The choice of this system was based on its advantages over other evidence grading systems, in particular because it allows the assessment of evidence quality to be separated from the assessment of the recommendation strength.

## RESULTS

**Figure 1:**
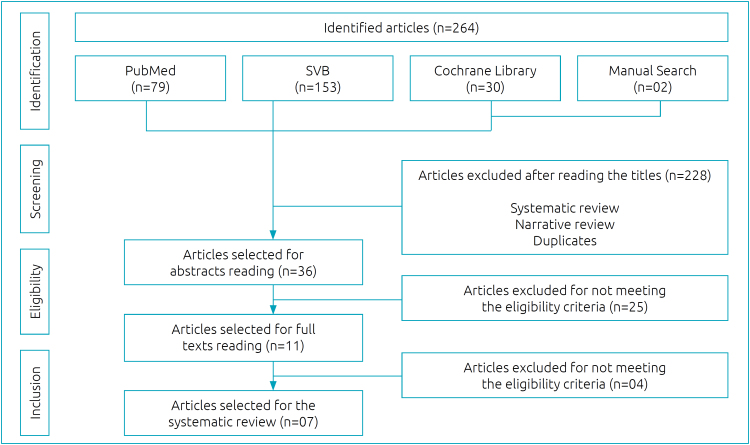
Flowchart of the selection of scientific articles.

A total of 264 articles were identified, of which 257 were excluded after applying the eligibility criteria and reading the abstracts and full texts. Thus, 7 studies were included in this review, being six observational studies and one experimental study. Total population consisted of 125 individuals, with samples ranging from 1 to 71 participants. Figure 1 shows the process of including studies in this review.

The selected studies were published between 1984 and 2022, most of them in the last 5 years (85.7%). As for the place where the studies were conducted, four of them (57.1%) were conducted in Brazil and the other three in Norway, Southeast Asia, and the United States. As for the language, 57.1% of the articles were published in English. Tables 2 and 3 present the summary of the papers included in this systematic review.

**Table 2:** Summary of the main descriptive characteristics of the studies published from 1984-2019 included in the Systematic Review.

Author	Study Design	Sample	Results
Goodman et al.^17^	Controlled trial, non-randomized	71 children with intellectual disabilities, at preschool age. Intervention Group: 35 children in hospital; 60.0% female, aged 33.7±9.8 months at first evaluation. Control Group: 36 children, 61.1% female, aged 37.7±10.4 months at the first evaluation.	Before the intervention the children had an average of 55.6±14.2 IQ points, while those in the control group had 59.3±13.0 points. After an average period of 16 months of intervention, there was a gain of 8.1 IQ points among the children in treatment compared to 0.8 points in the control group. An influence of the environment on the children’s growth was noted. It was shown that the stimulations performed by professionals need to be continued by the family. Moreover, individual characteristics have a great interference in the outcome of early stimulation, reinforcing the individuality of the treatment adapted to each case.
DeLuca et al.^14^	Case series	03 children (Child A, 54 months; Child B, 89 months; and Child C, 24 months), all with global developmental delays (limitations in fine/coarse motor skills, speech, communication, social interaction, and cognition).	Child A: Speech was stereotyped and repetitive, was unable to choose shapes, colors, and animals correctly as requested, although able to imitate spoken names. Followed one-step instructions and imitated short sequence behaviors. Child B: Had limited speech, identified colors and some animals, little environmental awareness, exhibited emotional outbursts. He was able to use the pencil, but not to make simple drawings, and the same happened with the use of scissors. Child C: Did not speak and presented fine and gross motor delays, was unable to sit up by himself or to make the transition to sitting in a typical manner for his age; he would not crawl. There was an increase in the development of each child (14 months increase for child A, 39 months for child B, and 20 months for child C), suggesting a good response to intensive therapy aimed at increasing functional skills/independence.
Silva et al. ^16^	Experience report	30 children with CZVS, aged 9 to 18 months, in Recife, Pernambuco, Brazil.	The children did not sit, hold their heads, or socialize. Some children were already able to maintain cervical tone and made postural changes. The study demonstrated the relevance of the cultural and social context for children with CZVS, highlighting the importance of real, contextual, and interpersonal interactions, facilitating the stimulation of skills such as sitting and standing prone, helping in cervical control and postural changes.
Monteiro et al.^20^	Controlled trial, crossover	16 children diagnosed with CZVS, 58.3% female, aged 23.9±3.97 months, with a birth head circumference of 28.6±1.34 cm, who were being followed at least once a week during the study period.	When analyzing moments T0 and T1, it was observed that after PI there was a significant decrease in the degree of tonus for the extensor muscles of the elbow (p=0.04) and knee (p=0.03). When comparing the interventions (PI versus PII), it was observed that in PI there was a reduction in the degree of tone for the extensor muscles of the elbow (p=0.03). In addition, after PI there was a significant reduction in the ranges of motion for elbow extension (p=0.05) and knee extension (p=0.03). When comparing the interventions (PI versus PII), statistical differences were observed between the ranges of motion for elbow extension (p=0.04), knee extension (p=0.05), and knee flexion (p=0.01). Regarding the level of stress during interventions, none of the 2 protocols promoted significant change.

IQ: intelligence quotient; CZVS: Congenital Zika Virus Syndrome; PI: Protocol 1; PII: Protocol 2; T0: time before intervention; T1: time immediately after intervention.

**Table 3:** Summary of the main descriptive characteristics of the studies published in 2020 and 2021 included in the Systematic Review.

**Amundsen and Evensen** ^18^	Case report	01 17-month-old child with CZVS, born in Southeast Asia, at 40 weeks, weighing 2,800 grams, 49 cm long, and a head circumference of 28.5 cm.	All activities were limited due to severe spasticity, and she had no voluntary movements. The child was uncomfortable, she cried a lot, and had difficulty feeding and sleeping. Initial physical therapy sessions were home-based and focused on spasm reduction, good resting positions, and parental support. At one year of age, postural control had already improved, leading to work on postural control of the head. The child showed an increased level of postural ability in the prone position and postural alignment; great gains in head and trunk alignment; improved general mobility and social skills.
**Tavares et al.** ^21^	Case report	01 child with mild microcephaly, diagnosed at 3 months of age, male, white, 2 years and 3 months old, 83 cm tall and weighing 10.2 kg, born with 35 cm of cephalic perimeter and presenting developmental delay.	He was unable to maintain control of the cervical spine, to manipulate objects and bring his hands to his mouth, would not roll over, had no alignment of head with midline and limbs, and could not perform any position progression. The patient also presented with marked choreoathetosis, with jerky movements, distortions, slowness, and incoordination in execution. At the end of the third week of care, the patient presented good cervical control, sustained for 50 seconds, hardly any falls, keeping the head aligned with the midline, with the body and limbs, brought the hand to the mouth very easily, and actively manipulated objects. In the final evaluation of the fourth week, compared to the previous weeks, the patient showed satisfactory results, with better cervical control, sustained for 1-3 minutes and no falls, showed head alignment with the midline, body and limbs, took his hand to his mouth easily, actively manipulated objects, started to crawl on the bed.
**Gama et al.,** ^22^	Retrospective Cohort	7 children, 2 of whom were female and 5 male; 3 of whom were born preterm, with the majority (n=5) from cesarean sections.	The children started stimulation therapy with a mean of 37.0±33.48 points on the GMFM scale. 188 sessions were scheduled, with 27±13.2 absences. The average gain of points in the GMFM questionnaire was 29.4±34.36 points and increased to 66.42±64.91 points. In some children, a stabilization of the gains or even a regression of the global scores was observed after the fourth week of intervention. Child #4 stood out in the improvement of motor function. We noticed a great variation in the GMFM score among the children, which shows the individuality of each one and the non-similar response to the treatment, which may be related to the neurological damage or to the number of sessions. The benefits of this type of therapy have been confirmed, even with severe damage to the central nervous system.

CZVS: Congenital Zika Virus Syndrome; GMFM: Gross Motor Function Measure.

**Figure 2:**
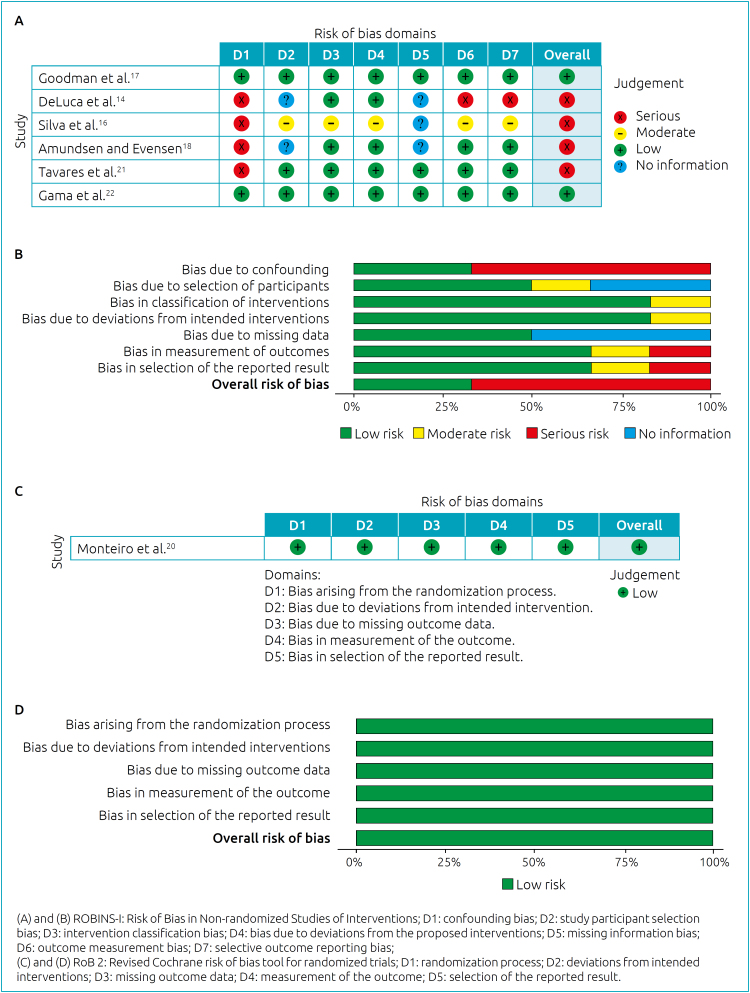
Individual risk and qualitative assessment of the risk of bias of the studies included in the systematic review.

The individual risk of bias assessment of the observational and experimental studies included in this systematic review are presented in Figure 2.

The assessment of the quality of evidence for the outcomes analyzed, considering the studies included in this systematic review, is shown in Table 4. After analysis using the GRADE system, it was observed that there is low confidence in the estimated effects, indicating the need for randomized clinical trials that can modify this.

**Table 4:** Quality of evidence of the outcomes evaluated.

Outcome	No. of participants of the included studies	Quality of evidence (GRADE)
Muscle tone	25	Low
Social interaction	31	Low
Fine and gross motor skills	42	Low
Intelligence quotient	71	Low
Socioemotional and adaptive behavior	54	Low

GRADE: Grading of Recommendations Assessment, Development and Evaluation

## DISCUSSION

This systematic review confirmed the existence of low-quality evidence regarding the impact of early intervention on the neuropsychomotor development of children with microcephaly, specifically on the outcomes of muscle tone, social interaction, fine and gross motor skills, intelligence quotient, language, and socioemotional and adaptive behavior.

Despite the paucity of evidence, considering that children with microcephaly face psychomotor, cognitive, and communicative impairments throughout their lives,^14^ the literature suggests that rehabilitation with accessible therapies can and should be used, as they stimulate the development of new functionalities. However, it is important to adapt these therapies to the age of the child in order to respect the limitations of each developmental stage and thus prevent accidents.^10^

In children with microcephaly, it is suggested that early rehabilitation be initiated during the period of greatest development, favoring greater neuroplasticity in the first three years of life, this being a critical period for child development.^15^ In addition to therapies, the involvement and commitment of the entire family is an unconditional part of the treatment, as well as the performance of a multidisciplinary team for proper monitoring and guidance.^16–19^

Among the studies analyzed, the main interventions performed were playful activities involving the use of body positions that stimulate specific muscle groups,^14,16,18,20,21^ either through stretching, physiotherapy equipment or aquatic activities. It has been found that the environment in which the child with microcephaly is inserted and the participation of the family in stimulation activities have advantages in the continuity of treatment and in understanding its importance.^16–18^

It is important to emphasize that some studies found in the literature analyzed mothers’ perceptions of the practice and importance of early stimulation for children with microcephaly, addressing the family’s perception of the needs and specificities of these children. In fact, however, the effects and effectiveness of the treatment used were not studied, which led to the exclusion of these articles from the present systematic review.

In the context of the outcomes examined in this review, Goodman et al.^17^ observed significant IQ gains in children with microcephaly who received early stimulation, demonstrating that the environment affects the growth of these individuals. DeLuca et al.,^14^ although describing the development of children with microcephaly not due to congenital Zika virus syndrome (CZVS), demonstrated significant gains in developmental milestones.

In addition, Silva et al.^16^ showed gains in muscle tone and socialization with the environment by involving the family of children with microcephaly in a carnival activity that included colors and textures, different tastes and temperatures, a decorated environment, and different positions as forms of stimulation. This finding corroborates the study by Monteiro et al.,^20^ who used the aquatic environment as a means of stimulation for children diagnosed with CZVS and showed a reduction in muscle tone and improvement in range of motion. It is important to note that the study by Monteiro et al.^20^ was the only randomized clinical trial that met the eligibility criteria established in this systematic review.

Among the studies reviewed, Amundsen et al.^18^ reported the use of early stimulation in a five-month-old child with parental participation in the activities. The authors described gains in postural ability, head and trunk alignment, general mobility, and social interaction.^18^ Tavares et al.,^21^ using intensive physical therapy in a child with mild microcephaly, showed improvements in neck control, head alignment, and manipulation of objects by placing them in the mouth.

In addition, Gama et al.^22^ followed-up a retrospective cohort of seven children who underwent intensive physiotherapy for one year and showed an increase in scores before and after the protocol in dimensions A (lying down and rolling over) and B (sitting), minimizing motor sequelae and maintaining acquired abilities. However, we noticed a large variability between the results of each child,^22^ which suggests the need for new studies considering larger samples.

Despite the positive results presented by the studies analyzed, from the improvement of neck support, gross motor skills and social interaction, a great variability of results was evident. This behavior may be related to the different levels of neurological damage, as well as the diversity of protocols applied. It is relevant to highlight the importance of classifying children diagnosed with mild and severe microcephaly, allowing the individualization of treatment for each functional alteration presented.

In addition, some of the studies carried out in Brazil point to the logistical difficulties of including these children in scheduled sessions, which may affect the progress and continuity of therapy.^20,22^ This finding reinforces the importance of involving parents and caregivers in the process of adherence and continuity of therapy.^20^

In general, the benefits of early stimulation therapy for human neurodevelopment have been demonstrated.^14,16–18,20–24^ However, there is a paucity of studies analyzing the appropriateness of available therapies applied to healthy children to the needs of children born with microcephaly.

Studies have shown that children affected by microcephaly due to maternal Zika virus contamination have severe motor impairments with significant delays in neuropsychomotor development.^25^ However, despite these findings, few studies have proposed to evaluate the efficacy of early stimulation in neuropsychomotor development of these children using appropriate methodological designs.

Therefore, as a potential limitation of the present study, systematic reviews of observational studies generally have weaker evidence than reviews of randomized clinical trials. However, in the absence of adequate clinical trials to evaluate the research question proposed in this study, the analysis of evidence from observational studies is justified.

In conclusion, this systematic review demonstrated the existence of low-quality scientific evidence regarding the influence of early intervention in the outcomes of muscle tone, social interaction, fine and gross motor skills, intelligence quotient, and socioemotional and adaptive behavior in children with microcephaly. The need for new randomized clinical trials is evident, as well as studies with low risk of bias and larger samples, investigating the effect of early stimulation on the neuropsychomotor development of these children.
